# Characterization of Adult Functional Traits of Local Populations and Cultivars of Sandberg Bluegrass and Bottlebrush Squirreltail Perennial Bunchgrasses

**DOI:** 10.3390/plants8060166

**Published:** 2019-06-11

**Authors:** Juan K. Q. Solomon

**Affiliations:** Department of Agriculture, Veterinary & Rangeland Sciences, University of Nevada, Reno, NV 89557, USA; jsolomon@cabnr.unr.edu

**Keywords:** Bottlebrush squirreltail, Sandberg bluegrass, total root length, root diameter, root carbon and nitrogen content, forage nutritive value

## Abstract

Plant functional traits offer an understanding of the plant’s ability to cope with varying environmental impositions. The objective of this study was to evaluate the above and belowground adult morphological and chemical composition traits of local populations of Sandberg bluegrass (*Poa secunda* J. Presl) and Bottlebrush squirreltail (*Elymus elymoides* (Raf.) Swezey) collected in Nevada and their cultivated varieties. A total of six replications (one seedling each) from each population and cultivar of the two native perennial bunchgrasses were used in a randomized complete block design experiment. Each of the six seedlings from each sourced population was transplanted into individual tree pots (28 cm diameter × 61 cm height) containing 20.4 kg of air-dried Orr gravelly sandy loam soil in mid-November, 2015 and remained in the pots for the duration of the study (23 June, 2016). Traits evaluated were, plant height, leaf length, inflorescence length, shoot biomass, forage nutritive value, root morphological traits, and root carbon and nitrogen content. Traits means were considered different at *P* < 0.05. For Sandberg bluegrass, the cultivar ‘Mountain Home’ and the population from Panther Valley tended to have greater biomass than the population from Button Point but overall, the average of the two cultivars (10.8 g/plant) did not differ in shoot biomass relative to the local populations (7.6 g/plant). For squirreltail, plant height for the George St. Sonoma and Grass Valley populations (71.3 cm) was greater than the cultivars ‘Toe Jam Creek’ and ‘Vale’ (40.5 cm) but cultivars had greater biomass (12.6 g/plant) than the local populations (5.8 g/plant). Total root length and root diameter were not different among the Sanberg bluegrass and squirreltail populations. The results from traits expounded on in this study indicate the closeness of these populations for both species at their adult stage and provide insights for building a unified framework approach among the different agencies and restoration practitioners to aid in plant assemblages for restoration success in the Great Basin and beyond.

## 1. Introduction

Restoration of habitats in the Great Basin of the Western United States has been an ongoing activity [1,2,3], mainly because of the frequency and intensity at which the native ecosystems are degraded. Factors that are responsible for the degradation and loss of habitats in the Great Basin are well known for example, climate change and the associated increases in drought, the propensity at which habitats are occupied by fire-prone nonnative annual grasses, for example, cheatgrass (*Bromus tectorum* L.) and medusahead (*Taeniatherum caputmedusae* [L.] Nevski), the spread of noxious weeds and their ability to dominate native vegetation, increased fuel properties resulting in wildfires [2,4,5,6,7], and unsustainable domestic livestock grazing management practices [8,9,10]. These aforementioned factors are considered among the foremost that threaten the ecological stability in the Sagebrush steppe ecosystems in the Western United States. Further, these changes threaten the existence of important flora needed for ecological resiliency and wildlife habitat for occupancy and feed of endangered fauna, for example, greater sage-grouse (*Centrocercus urophasianus*) in the Western United States [5,11].

The wildland fires during the summers of 1999 and 2000 that burned approximately 2.4 million acres in the Great Basin, triggered a concerted effort by concerned institutions and interest groups that led to the formation of the Great Basin Restoration Initiative to highlight the plight and curtail the ecological misfortunes of the Great Basin [12]. The Great Basin Restoration Initiative (GBRI) emphasized that there must be greater use of native species in restoration efforts of degraded plant communities in the Great Basin particularly those plants that can survive in competition with weeds [12]. Numerous other reports have endorsed this approach of using locally-adapted native species because of their known ecological fitness for the intended sites [13,14,15,16]. However, restoring diverse native plant communities is not a trivial exercise in nature [17], because of the multiplicity of factors that are known to create hindrances to the successful restoration of degraded plant communities, more so in semiarid and arid ecosystems. One such factor that hinders restoration efforts is the limitation of our understanding of functional traits associated with native bunchgrasses.

Plant functional traits offer an understanding of the plant’s ability to cope with varying environmental impositions. For example, the tall species of native grasses invest in structural biomass which improves their light capturing ability [18] and overall biomass productivity. Native perennial grasses with greater shoot biomass have been linked as a valuable trait to defoliation tolerance [19]. There is a direct correlation between the inflorescence axis of plants and seed mass [20], thus increasing the plant’s ability to survive and compete with invading species [21]. Further, plants with large root systems have a competitive advantage for nutrient and water uptake [22] compared to those with small root systems. For example, plants with greater root biomass allocation were reported by Ledger and Baughman [23] to increase native perennial grass performance in the field. Root morphological parameters, namely root length, root diameter, root branching, root weight, root volume, root-to-shoot ratio, and specific root length are all associated with the increased performance of native plants under drought and resource-limited soil conditions [23,24,25,26,27]. Traits prioritized in native plant materials and cultivars were valued in the following order of forage quality and yield, seed yield, ability to establish and persist, and drought tolerance [23]. Both forage quality and yield are linked to aboveground morphological and chemical composition traits and the latter two are linked to belowground morphological traits. Intraspecific comparisons are most valuable in the characterization of native plant materials for restoration [28] because of the variations among ecotypes of the same species [29]. Variation in native plant materials genetics and cultivated varieties can alter plant success in restoration effort [30]. Therefore, an understanding of these plant functional traits provides a useful tool for predicting the effects of herbivory and other disturbances on ecosystems resilience [10]. 

Knowledge of belowground partitioning of native plants is strongly linked to the trait–environment relationships [20] and have valuable implications for restoration success. There is information available for native perennial bunchgrasses traits, but largely at the seedling stage for local ecotypes collected in Nevada [31,32]. However, there is a dearth of information on adult functional traits of native bunchgrasses ecotypes present in Nevada. Therefore, seed source selection can be refined by the characterization of these traits among native perennial bunchgrasses populations in Nevada. Understanding the variations in adult functional traits among wildland populations of native perennial bunchgrasses will help us make prudent decisions in plant assemblages for restoration success [33] and sustenance of vigorous ecosystems in the Great Basin and beyond. The hypothesis of this study was that released cultivars of both Sanberg bluegrass (*Poa secunda* J. Presl) and Bottlebrush squirreltail (*Elymus elymoides* [Raf.] Swezey) native perennial bunchgrasses will have greater aboveground morphological traits (plant height, leaf length, tillers per plant, inflorescence length, and shoot biomass), aboveground chemical composition traits (crude protein, neutral detergent fiber, acid detergent fiber, acid detergent lignin, ash, and digestibility), belowground root morphological traits (root length, root diameter, and root volume), and root carbon and nitrogen concentrations than their local ecotypes. The objective of this study was to evaluate the above and belowground adult morphological and chemical composition traits of populations of Sandberg bluegrass and Bottlebrush squirreltail perennial bunchgrasses collected in Nevada and their cultivated varieties.

## 2. Results

### 2.1. Sandberg Bluegrass Aboveground Traits

**Figure 1:**
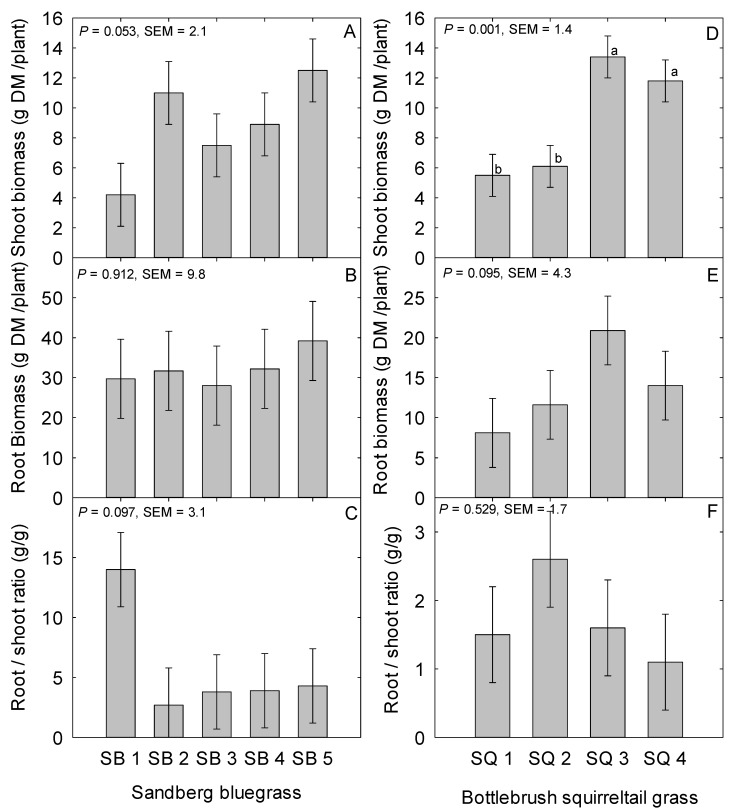
Mean shoot biomass dry matter (DM) grams per plant (**A**,**D**), root biomass DM grams per plant (**B**,**E**), root-to-shoot (gram root per gram shoot biomass DM) biomass ratio (**C**,**F**) of Sandberg bluegrass and Bottlebrush squirreltail populations evaluated under greenhouse conditions during 2015 to 2016 at the University of Nevada, Reno, USA. SB 1; Button Point (LP), SB 2; Panther Valley (LP), SB 3; Winnemucca Mountain (LP), SB 4; Hanford (CV), SB 5; Mountain Home (CV), and Bottlebrush squirreltail, SQ 1; George St. Sonoma (LP), SQ 2; Grass Valley (LP), SQ 3; Toe Jam Creek (CV), and SQ 4; Vale (CV). LP; local population, and CV; cultivar. Bars with same lowercase letters are not different (*P* > 0.05).

Among the Sandberg bluegrass populations, plant height and tillers/plant were not different (Table 1). The average plant height and tillers/plant for the Sandberg bluegrass populations evaluated were 22.7 cm (SEM = 1.7) and 262.8 (SEM = 33.0) respectively (Table 1). Plant height did not differ (*P* = 0.552; SEM = 1.2) between cultivar (23.3 cm) compared to the local population (22.3 cm) when averaged across category. However, leaf length was different (*P* = 0.032) among Sandberg bluegrass populations (Table 1). The Sandberg bluegrass cultivar Hanford had longer leaves than the local population from Button Point (*P* = 0.002) and the cultivar Mountain Home (*P* = 0.036) (Table 1). Further, the population from Panther Valley had longer leaves (*P* = 0.049) than the one from Button Point (Table 1). There was no difference (*P* = 0.112) in leaf length between the averaged of cultivars and the local populations. Pertaining to shoot biomass, there was a trend for a population effect (*P* = 0.053; Figure 1A). The cultivar Mountain Home (*P* = 0.006) and the population from Panther Valley (*P* = 0.021) tended to have greater biomass than the population from Button Point (Figure 1A) but overall, the average of the two cultivars (10.8 g/plant) did not differ (*P* = 0.119; SEM = 1.7) in shoot biomass relative to the average of local populations (7.6 g/plant).

### 2.2. Sandberg Bluegrass Belowground Traits

**Figure 2:**
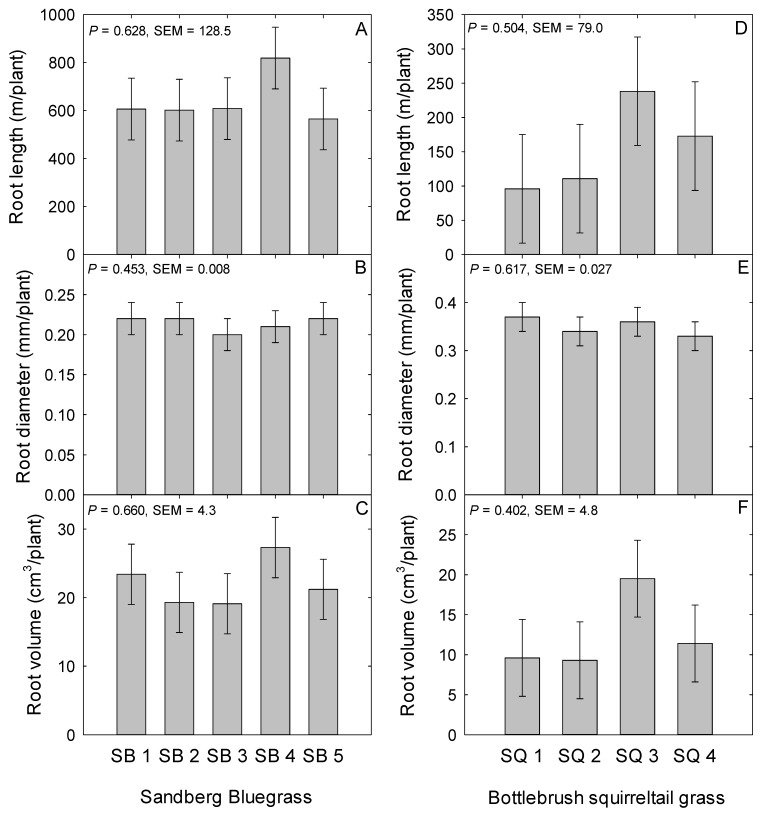
Mean (**A**,**D**) total root length in meters, (**B**,**E**) root diameter in millimeters, (**C**,**F**) root volume in cubic centimeters per plant of Sandberg bluegrass and Bottlebrush squirreltail evaluated under greenhouse conditions during 2015 to 2016 at the University of Nevada, Reno, USA. SB 1; Button Point (LP), SB 2; Panther Valley (LP), SB 3; Winnemucca Mountain (LP), SB 4; Hanford (CV), SB 5; Mountain Home (CV), and Bottlebrush squirreltail, SQ 1; George St. Sonoma (LP), SQ 2; Grass Valley (LP), SQ 3; Toe Jam Creek (CV), and SQ 4; Vale (CV). LP; local population, and CV; cultivar.

In relation to root morphological traits, TRL, RD, and RV were not different among the Sanberg bluegrass populations or between the cultivar and local population comparison (*P* > 0.05; Figure 2A–C). Average TRL and RD among Sandberg bluegrass populations were 640 m (SEM = 128.5) and 0.21 mm (SEM = 0.01) respectively (Figure 2A,B). Root biomass was not different among the Sandberg bluegrass populations (*P* = 0.912; Figure 1B). The specific root length (SRL) did not differ (*P* = 0.556; SEM = 7.4) among populations nor between the contrast (*P* = 0.344; SEM = 5.2) of cultivar (17.7 m/g) versus the local population (24.1 m/g) of Sandberg bluegrass. For the root-to-shoot biomass ratio (RSBR), there was a trend for a population effect (*P* = 0.097; Figure 1C). The RSBR of the Button Point population tended to be greater than the four other populations (Figure 1C). For the average of the two categories, RSBR was not different (*P* = 0.395; SEM = 2.3) between cultivar (4.6 g/g) and the local population (6.7 g/g) of Sandberg bluegrass.

### 2.3. Sandberg Bluegrass Forage Nutritive Value

**Figure 3:**
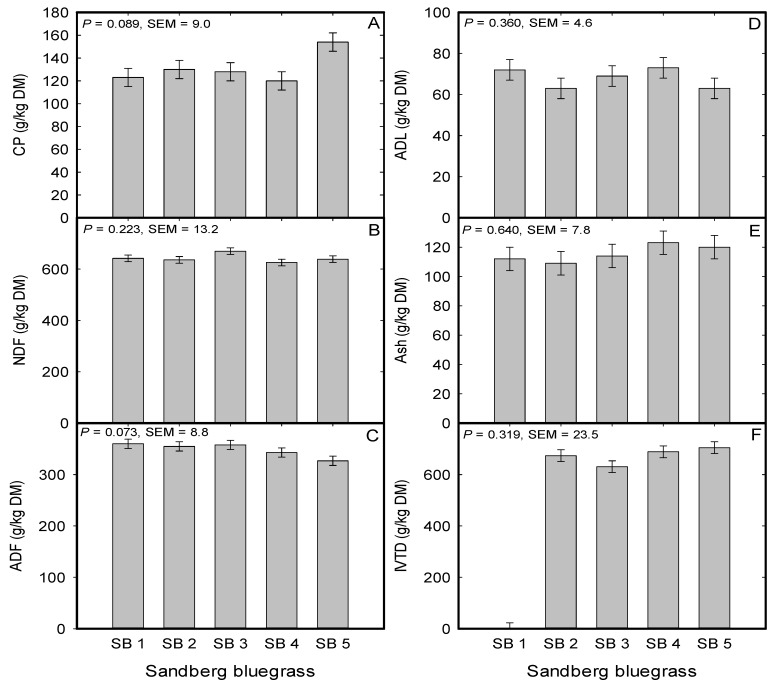
Mean (**A**) crude protein (CP), (**B**) neutral detergent fiber (NDF), (**C**) acid detergent fiber (ADF), (**D**) acid detergent lignin (ADL), (**E**) ash, and (**F**) in vitro true digestibility of Sandberg Bluegrass (expressed in gram per kilogram of dry matter (DM)) evaluated under greenhouse conditions during 2015 to 2016 at the University of Nevada, Reno, USA. SB 1; Button Point (LP), SB 2; Panther Valley (LP), SB 3; Winnemucca Mountain (LP), SB 4; Hanford (CV), SB 5; Mountain Home (CV). LP; local population, and CV; cultivar.

For forage nutritive value, there were trends for a population effect on CP (*P* = 0.08; Figure 3A) and ADF concentrations (*P* = 0.07; Figure 3C). For CP concentration, the cultivar Mountain Home tended to have greater concentration than the population from Button Point (*P* = 0.020) and the cultivar Hanford (*P* = 0.012) (Figure 3A). Pertaining to the ADF concentration, the cultivar Mountain Home tended to have lower ADF than the local populations from Button Point, Panther Valley, and Winnemucca Mountain (*P* = 0.022). However, the NDF, ADL, Ash, and IVTD concentrations were not impacted by Sandberg bluegrass populations (Figure 3B,D,E,F). Among the nutritive value parameters, when average across category (cultivar vs. local population), the ADF (*P* = 0.009; SEM = 6) and IVTD concentrations (*P* = 0.031; SEM = 10) were different between cultivar and the local population. For ADF, cultivar (336 g/kg) had less concentration than local population (357 g/kg) and for IVTD, cultivar (658 g/kg) had greater concentration than the local population (621 g/kg).

### 2.4. Bottlebrush Squirreltail Aboveground Traits

For squirreltail populations, plant height was different (Table 1). Plant height for the George St. Sonoma and Grass Valley populations was greater (*P* < 0.001) than that of the cultivars Vale and Toe Jam Creek (Table 1). Average plant height was greater (*P* < 0.001; SEM = 2.9) for the local population (71.3 cm) compared to cultivar (40.5 cm). The inflorescence length of squirreltail was affected by population (Table 1). The inflorescence length of the cultivar Vale was greater than the George St. Sonoma and Grass Valley populations also, the cultivar Toe Jam Creek had longer inflorescence than the George St. Sonoma population (Table 1). Further, inflorescence length was longer (*P* = 0.003; SEM = 0.94) for cultivar (14.8 cm) than the local population (10.2 cm). There was a trend (*P* = 0.064) for a population effect on leaf length (Table 1). The cultivar Toe Jam Creek tended to have longer leaves (*P* = 0.012) than the Grass Valley population (Table 1) and also a trend (*P* = 0.086; SEM = 0.83) for longer leaves for cultivar (18.7 cm) compared to the local population (16.8 cm). There was no effect of squirreltail population on tiller/plant (Table 1) and the contrast between cultivar and local population (*P* = 0.894). The average number of tillers/plant was 143 across populations (Table 1). In relation to shoot biomass, there was a population effect (*P* = 0.001; Figure 1D). The two cultivars (Toe Jam Creek and Vale) had greater biomass than the two local populations collected from George St. Sonoma (*P* = 0.007) and Grass Valley (*P* = 0.001) (Figure 1D).

### 2.5. Bottlebrush Squirreltail Belowground Traits

Similar to the Sandberg bluegrass populations, the root morphological traits, (TRL, RD, and RV) were not different among the squirreltail populations (*P* > 0.05; Figure 2D–F) nor between cultivar versus local population average (*P* > 0.05). The average TRL and RD for squirreltail populations were 145.4 m and 0.35 mm, respectively (Figure 2D,E). There was a trend for a population effect (*P* = 0.095) on root biomass of squirreltail (Figure 1D). Root biomass of the local collection from George St. Sonoma tended to be less (*P* = 0.017) than the cultivar Toe Jam Creek in this study (Figure 1E). Root biomass was greater (*P* = 0.041; SEM = 3.6) for cultivar (17.4 g) compared to the local population (9.8 g). Squirreltail populations also did not differ (*P* = 0.447; SEM = 9.1) in SRL or the contrast (*P* = 0.195; SEM = 7.8) between cultivar (15.8 m g^−1^) and local population (24.4 m g^−1^). Neither squirreltail population or the contrast between cultivar and the local population had an effect (*P* = 0.529) on the RSBR in this study (Figure 1F).

### 2.6. Bottlebrush Squirreltail Forage Nutritive Value 

**Figure 4:**
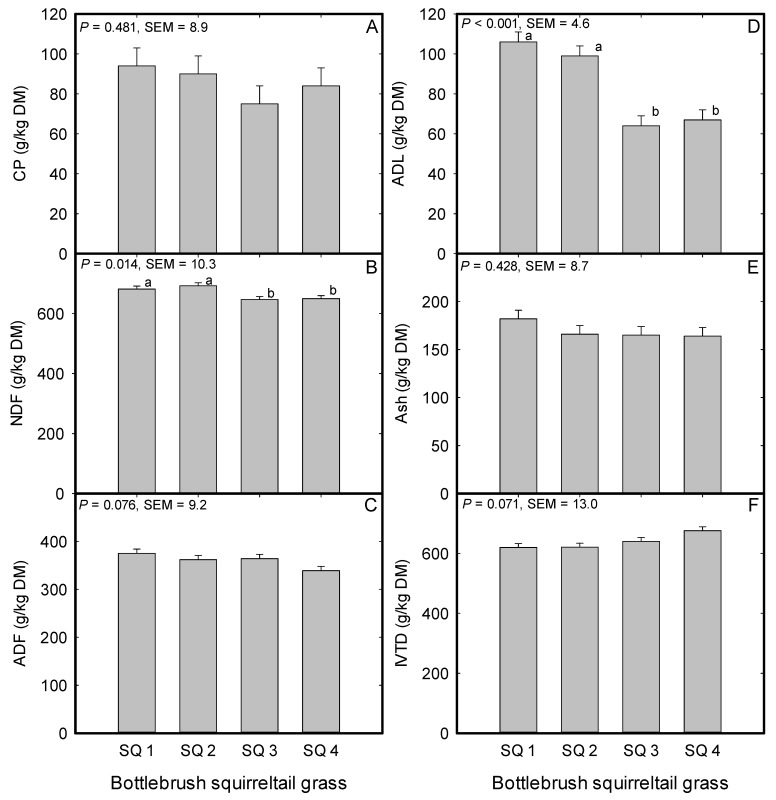
Mean (**A**) crude protein (CP), (**B**) neutral detergent fiber (NDF), (**C**) acid detergent fiber (ADF), (**D**) acid detergent lignin (ADL), (**E**) ash, and (**F**) in vitro true digestibility of Bottlebrush squirreltail (expressed in gram per kilogram of dry matter (DM)) evaluated under greenhouse conditions during 2015 to 2016 at the University of Nevada, Reno, USA. SQ 1; George St. Sonoma (LP), SQ 2; Grass Valley (LP), SQ 3; Toe Jam Creek (CV), and SQ 4; Vale (CV). LP; local population, and CV; cultivar. Bars with same lowercase letters are not different (*P* > 0.05).

There were population effects on NDF (*P* = 0.01) and ADL (*P* = 0.001) concentrations and trends for population effects on ADF (*P* = 0.07) and IVTD (*P* = 0.07) concentrations (Figure 4). However, the CP and Ash concentrations were not different among squirreltail populations (*P* = 0.48; 0.43 respectively, Figure 4). For NDF concentration, the two cultivars (Toe Jam Creek and Vale) had lower concentrations (*P* = 0.023) than the two local populations (George St. Sonoma and Grass Valley) in this study (Figure 4B). The ADF concentration tended to be less for the cultivar Vale (*P* = 0.013) compared to the population from George St. Sonoma (Figure 4C) and for IVTD, the cultivar Vale tended to have greater IVTD concentration than the George St. Sonoma (*P* = 0.023) and Grass Valley populations (*P* = 0.026) (Figure 4F). For the contrast between cultivar and local population of squirreltail, only NDF (*P* = 0.001; SEM = 7.0) and ADL concentrations (*P* < 0.001; SEM = 3.0) were different. The NDF concentration for cultivar (649 g/kg) was less than the local population (648 g/kg). While for ADL, cultivar (65 g/kg) had less concentration than the local population (103 g/kg) average. There was also a trend (*P* = 0.099; SEM 7.0) for cultivar to have less ADF concentration (351 g/kg) than the local population average (369 g/kg) in this study.

## 3. Discussion

The hypothesis of this study was that released cultivars of both Sanberg bluegrass (*Poa secunda* J. Presl) and bottlebrush squirreltail (*Elymus elymoides* [Raf.] Swezey) native perennial bunchgrasses have greater aboveground morphological traits (plant height, leaf length, tillers per plant, inflorescence length, and shoot biomass), aboveground chemical composition traits (crude protein, neutral detergent fiber, acid detergent fiber, acid detergent lignin, ash, and digestibility), belowground root morphological traits (root length, root diameter, and root volume), and root carbon and nitrogen concentrations than their local ecotypes. The findings in this study partially supported the hypothesis in relation to the aforementioned traits differences between released cultivars and local populations of the two native perennial bunchgrasses appraised under greenhouse environment. The characterization of adult functional traits of native perennial bunchgrasses has direct implications in elucidating their suitability as compatible and companion species in mixed plant communities through functional complementarity [34,35], and aid in the formation of stable plant communities through prudent plant species assemblage based on the ecosystem to be restored [23,36,37]. This approach will possibly enable plants to cope with both biotic and abiotic stresses that affect plant development, morphology, and fitness [38]. Further, it will guarantee some desired level of stable primary productivity [39] due to their differential resilience of the different plant species which will support wildlife sustenance coupled with other ecosystem services [40]. Success in restoration goes beyond the plant functional traits with differing views globally [41,42]. However, this trait-based approach coupled with an understanding and remedies for interacting factors of landscape structure, edaphic, biological, climate change, and organisms beyond plants (e.g., soil crust restoration, soil-atmosphere exchanges) will enhance success and stability of the global degraded ecosystems [42,43,44,45].

In this study, the cultivar Hanford had longer leaves relative to the cultivar Mountain Home and the local population from Button Point respectively. Additionally, the wildland population from Panther Valley leaves were longer than the population collected from Button Point. In support of the leaf length results among Sandberg bluegrass populations, Johnson et al. [40] reported that leaf length differed among sourced populations of Sandberg bluegrass and when differences occurred, cultivars generally had longer leaves than wild populations. Native perennial bunchgrasses with longer leaves may affect the ability of companion flora of similar growth habit to stride well in mixed plants communities because of their morphological dominance thereby suppressing companion species through shading [46,47,48]. However, the advantage concerning perennial native bunchgrasses with longer leaves can be seen as a positive because they may be able to outcompete annual invasive such as cheatgrass (*Bromus tectorum* L.) [49], thus adding stability to these highly competitive and unstable grassland ecosystems of the Western United States. It is also possible that the advantage of longer leaves observed in this study for cultivars, do not carry any meaningful biological significance in the form of morphological dominance, or self-shading thereby, allowing for suitable compatibility and adaptability at the intended restoration site. Depending on leaf orientation, plants with longer leaves can first create impediments to its own self by self-shading [50] thereby, reducing light interception at the lower canopy level and thus resulting in lower overall photosynthetic activity, and ultimately productivity [51,52].

In this study, the mean leaf length of Mountain Home (15 cm) and plant height (22.9 cm) were similar to the leaf length (5 to 15 cm) but different than the mean plant height (40 cm) in the morphological description of Mountain Home release [30]. Further, this variation in plant height may indicate the morphological plasticity of these grasses [53,54] depending on environmental conditions at the site of cultivation. Unlike this study, Shaw and Mummey [55] and Johnson et al. [40] reported distinct evidence of genetic variations among populations of Sandberg bluegrass across the Intermountain West in the United States. The disparity in this study relative to the study of Johnson et al. [40] is that only a small number of sourced populations of Sandberg bluegrass were evaluated.

Pertaining to plant dry matter production, Herget et al. [56] reported a unique trend in their study that biomass production was greater for cultivars of Sandberg bluegrass relative to those of the wild accessions but when they removed the ‘High Plains’ cultivar, biomass production was somewhat similar between cultivated and wild accessions a trend similar to the results observed in this study. The trend for differences in shoot biomass in this study, may be partially explained by the differences in leaf length as there was a positive association between shoot biomass and leaf length (*r* = 0.60; *P* = 0.001) similar to the results of Arrendondo et al. [57] and possibly as a result of greater photosynthetic rate for longer leaves [58] in this study. No other meaningful correlations occurred among variables measured for Sandberg bluegrass populations. Additionally, in contrast with this study, Johnson et al. [40] reported that dry shoot weight was greater for cultivars (26.9 g/plant) compared to wild populations (20.0 g/plant) unlike the results of our study of lack of difference in dry shoot weight when cultivar was contrasted to the local population. This common trend of greater biomass production for cultivar versus non-cultivar may indicate cultivar vigor [59,60] and thus, possible dominance in restored vegetation limiting the diversity of plant community structure [61]. However, this biomass indicator was not distinct among populations of Sandberg bluegrass in this study and thus, less conclusive evidence supporting the cultivar vigor hypothesis [61]. Overall, Sandberg bluegrass plant dry shoot weight in this study ranged from 4.2 to 12.5 g/plant and was somewhat lower than those reported by Johnson et al. [40].

Contrary to the Sandberg bluegrass populations, there were distinct differences among squirreltail populations in the aboveground morphological traits of plant height, inflorescence length, and shoot biomass that supported the hypothesis of this study (Figure 1/Table 1). In concurrence with this study, Jones et al. [36] in a trial that lasted for 34 days under greenhouse conditions reported significant variation in leaf length and total plant dry matter among *Elymus elymoides* accessions. Further, Parsons et al. [28] reported a significant effect of squirreltail sourced population on the measured traits of leaf length, canopy height, tiller number, and dry shoot mass under greenhouse house and field evaluations. This study showed a trend for leaf length difference that favors the cultivar Toe Jam Creek by 4.5 cm over the wildland population from Grass Valley. Although squirreltail populations were different in biomass and greater for cultivars by an average 117.2% relative to the wild populations, tillers/plant, a key growth unit of grasses linked to biomass production [62] were not different in this study. Further, contrary to biomass production, plant height on average was 31.1 cm taller for the two locally sourced populations (George St. Sonoma and Grass Valley) relative to the two cultivars (Toe Jam Creek and Vale) and did not translate into greater biomass in this study. The inflorescence was longer for both cultivars (Toe Jam Creek and Vale) relative to the local population from George St. Sonoma by 6.15 cm and 4.3 cm respectively. Based on correlation analysis in this study, tiller number was not different and did not correlate with shoot biomass (*r* = 0.14; *P* = 0.518) similar to the results of Parsons et al. [28]. However, plant height had a negative association with biomass (*r* = −0.507; *P* = 0.011) and was in contrast to the results reported by Parsons et al. [28]. In this study, inflorescence length was positively associated with biomass (*r* = 0.63; *P* = 0.001) and therefore, 63% of the variation in biomass can be attributed to the difference in inflorescence length between cultivars and wildland populations squirreltail. Tiller diameter and mass can influence biomass production of plants [28] and the shorter tillers for the two cultivars were perhaps denser than their elongated counterparts of the two local collections which may be another possible reason for the difference in biomass production in this study. Also, there was a positive correlation between shoot and root biomass (*r* = 0.45; *P* = 0.028) among squirreltail populations in this study, which was similar to the shoot and root biomass association reported by Parsons et al. [28]. The range of plant height 40.5 to 66.8 cm reported by Parsons et al. [28] was similar to the ranged recorded in this study (37.4 to 71.6 cm). Parsons et al. [63] in their study reported dry weight production per plant ranged from 8.9 to 31.7 g/plant in the first year, and from 5.8 to 67.7 g/plant in the second year, which demonstrates the considerable variation among squirreltail accessions. However, in this study biomass production range was less varied among squirreltail populations. Other contrasting observations in the study by Parsons et al. [28] relative to this study was the strong positive association between plant height and biomass (*r* = 0.643) and the tiller number after 60 d was significant among squirreltail accessions contrary to this study that lasted the full season and thus, much older plants. Therefore, these different scenarios of longer leaves impact along with the other aboveground morphological traits must be taken into consideration in the assemblage of native plants for restoration efforts globally in the context of the changing climate and drought-induced mortality [41,42].

A trait that is not widely studied among populations of native perennial bunchgrasses in the great basin of the Western United States is their nutritive value, which is an important component in wildlife and domesticated herbivores nutrition. Forage nutritive value is intricately linked to the health and performance of grazing herbivores through the provision of digestible energy, CP, minerals, and vitamins [64]. The results indicated trends for both CP and ADF concentrations difference between the cultivars and locally sourced populations. For example, the cultivar Mountain Home had 25.2% greater CP concentration than the local ecotype from Button Point and between the two Sandberg bluegrass cultivars, the CP concentration of the cultivar Mountain Home was 28.3% greater than the CP concentration of the cultivar Hanford. Both cultivars (Mountain Home and Hanford) had an average 5.4% lower ADF concentration than the local populations from Winnemucca Mountain, Button Point, and Panther Valley. However, the lower ADF did not translate into greater digestibility for the cultivars relative to the locally sourced populations of Sandberg bluegrass as is typically the trend in forage quality evaluations [65]. For the squirreltail populations, both local populations (George St. Sonoma and Grass Valley) had an averaged 5.7% greater NDF and a 36.1% greater ADL concentrations than the two cultivars (Toe Jam Creek and Vale). The 9.6% lower ADF for the cultivar Vale relative to the local population from George St. Sonoma translate into a 7.5% greater digestibility for Vale than the two local populations (George St. Sonoma and Grass Valley). In studies that evaluated nutritive value of Sandberg bluegrass, Cruz and Ganskopp [66] and Ganskopp and Bohnert [67] reported CP concentration range from 122 g/kg at vegetative stage to 88 g/kg at anthesis and for NDF concentration from 617 g/kg at vegetative to 605 g/kg at anthesis. In the same study by Ganskopp and Bohnert [67], the values for squirreltail were 179 g/kg CP at the vegetative stage to 98 g/kg at anthesis and for NDF concentration 542 g/kg at the vegetative stage to 630 g/kg at anthesis. This study was terminated at the anthesis stage and the nutritive value for CP and NDF were in line with those reported above. Further, Sandberg bluegrass ash concentration was similar to the 101 g/kg reported by Demarchi [68] and the 604 to 584 g/kg dry matter digestibility reported by Jefferies and Rice [69]. The nutritive value of the two native perennial bunchgrasses in this study was either superior or similar to the nutritive value of 14 rangeland grasses evaluated for winter forage by Jensen et al. [70] thus, indicating the value of these two native bunchgrasses as feed for domestic livestock, wildlife, gaming activities support, and must be taken into consideration in the assemblages of native plants for restoration of degraded landscapes [71].

Plant roots are vital in soil water and nutrient uptake, carbon and nitrogen storage for plant recovery post defoliation, and its role in ecosystem services e.g., carbon sequestration is well documented in grassland ecosystems [27,72,73,74]. An understanding of plant root morphological traits will lead to a better understanding of plant fitness [27] under varying environmental conditions, particularly the heterogeneous geomorphic nature of these ecosystems globally with increasing aridity [75]. Pertaining to belowground traits, apart from a trend for root biomass difference among squirreltail populations, neither of the two native perennial bunchgrasses populations differed in root morphological traits of TRL, RD, RV, SRL, and root biomass among Sandberg bluegrass populations. Unlike this study, Parsons et al. [28] in a 60-d greenhouse trial evaluating squirreltail, Atwater et al. [32] working with squirreltail 10-d glasshouse experiment, and Ferguson et al. [26] working with squirreltail in a 30- to 120-d greenhouse trial and Parsons et al. [28] in a greenhouse study that lasted 60-d using squirreltail found significant main effects of seed source on root morphological traits. These studies were done over a short duration (seedling stage), unlike this study which lasted a full growing season. Therefore, at the seedling stage, these variations may be a result of differences in seed mass and storage reserve providing an early advantage in root traits for populations with larger seed size [76,77] but at the adult stage, there was parity in belowground morphological traits, possibly because of similar photosynthetic activity. This indicates that seedling performance is not indicative of mature plant performance, as demonstrated by Parsons et al. [28]. In another study using cultivars relative to non-cultivars of *Sorghastrum nutans* and *Schizachyrium scoparium,* Klopf and Baer [59] found significant variation in root length, surface area, and root volume, and recommended that population source selection should be considered in setting restoration goals and objectives.

The RSBR allocation is an important trait and indicates the ability of plants to compensate for limited resources in their environment and therefore, survive and succeed in competition [23,78]. This study only revealed a trend for a difference in RSBR among Sandberg bluegrass sourced populations and no statistical difference among squirreltail populations. The RSBR favored the local population from Button Point relative to all other Sandberg bluegrass populations. This indicates that the sourced population from Button Point invested more in root than shoot biomass and therefore, may persist better in moisture-deficit and nutrient-poor soils. In complete contrast to this study, Jones et al. [36], Parsons et al. [28], Parsons et al. [63], and Rowe and Leger [79] have all reported distinct variation in RSBR among sourced populations of the squirreltail bunchgrass taxa (*Elymus* spp.). In the study by Parsons et al. [28] root mass was significant among populations and range from 33.3–95.6 mg/plant while the root-to-shoot ratio was also significant among accessions and ranged from 0.227–0.434 at 60-d growth duration in a greenhouse environment. However, the duration of these studies was short (25- to 100-d) and therefore, characterized at the seedling stage relative to this study of a full growing season. This is further confirmation that seedling traits may not necessarily be a good predictor of adult functional traits and subsequently the ability of the plant to survive and performed well in degraded ecosystems. Overall, based on the results of this study, the two native bunchgrasses invested more in root than shoot biomass [79] possibly because of evaporative demand in their area of origin that necessitate a greater RSBR [36].

Defoliation is a recurring factor that alters the vegetational state in grassland ecosystems [73]. The remobilization of C and N content in roots of grasses are important for compensatory photosynthesis and regrowth which may indicate plant species potential to recover post defoliation following the loss or severe reduction in photosynthetic activity [80,81,82]. Also, root carbon content indicates grassland ability to sequester carbon an important facet in greenhouse gas mitigation and ecosystem services [83]. This study revealed no statistical significance for both C and N concentration and content among Sandberg bluegrass populations which was similar to the results reported by Klopf and Baer [59]. For the populations of squirreltail evaluated, there was a trend for greater C (166.4% increase) and N (146.6%) content of the cultivar Toe Jam Creek relative to the local population from George St. Sonoma and this may indicate the potential for greater recovery of the cultivar post defoliation. This observed greater root C (148.6 g/plant) and N (5.7 mg/plant) content was attributed to the magnitude of difference in root biomass between the two populations rather than C and N concentrations [84]. Johnson et al. [40] working with *Poa secunda* populations attributed 77% of the phenotypic variation to seed source collection but in this study, both Sandberg bluegrass and squirreltail populations did not display wide variations in plant functional traits and this may indicate the close similarities of the local populations and the cultivated varieties for the two native bunchgrasses evaluated.

## 4. Materials and Methods 

### 4.1. Plant Collection and Study Location

Three locally sourced populations of Sandberg bluegrass (*Poa secunda* J. Presl) and two Bottlebrush squirreltail (*Elymus elymoides* (Raf.) Swezey) were collected from different ecoregions in Nevada during the period of May to June of 2015 (Table 2). The collection sites (Table 2) were selected because of their species richness and the most targeted areas by seed collectors in the state of Nevada. Two cultivars from each species were included in this study for a comparative assessment between local populations and their commercially released counterparts (Table 2). This greenhouse study was carried out in the University of Nevada, Reno Greenhouse Complex Reno, Nevada (39° 36′ 50.4576″ N, 119° 52′ 38.7228″ W). Sandberg bluegrass is a cool-season densely tufted native short-lived perennial bunchgrass indigenous to the Sagebrush steppe ecosystem of Western United States. It has been deemed a unique grass species because of its fixed adaptation [85] and its reproduction through facultative apomixis [86]. It is a source of valuable feed for livestock and wildlife but generally does not produce much useable forage because of its small stature and early maturity [87]. Bottlebrush squirreltail commonly called squirreltail is an indigenous cool-season perennial bunchgrass native to western North America [88]. It is a self-pollinating allotetraploid and is commonly hybridized with other *Elymus* spp. [88]. It is typically found at elevations ranging from 600 to 3500 m and has the potential to invade and outcompete invasive species such as cheatgrass and medusahead [57]. It is a valuable feed for domestic livestock and wildlife in the Western United States [89].

The ecological sites were within the Great Basin shrub steppe of Nevada and generally modified by mowing, burning, grazing, and invasion by cheatgrass (*Bromus tectorum* L.). Apart from the targeted species for collection, the sites were generally comprised of the following associated plants species; *Lepidium perfoliatum*, *Salsola tragus*, *Atriplex confertifolia*, *Artemisia spinosa*, *Chorispora tenella*, *Descurainia sophia*, *Sisymbrium altissimum*, *Erodium cicutarium*, *Artemisia tridentata* ssp. *wyomingensis*, *Ericameria nauseosa*, *Descurainia pinnata*, *Achnatherum hymenoides*, *Sarcobatus vermiculatus*, *Grayia spinosa*, *Sphaeralcea grossulariifolia*, *Alyssum desertorum*, and *Ceratocephala testiculata*.

### 4.2. Seedling Establishment and Transplant, Experimental Design, and Management

Seeds from each species collection were first seeded in late August of 2015 in cone-tainer tubes filled with Premier PRO-MIX growing medium that contained 75%–85% Canadian Sphagnum peat moss (Premier Tech Home and Garden, Quakertown, PA). In preparation for seedling transfer, the soil used was an Orr gravelly sandy loam (Fine-loamy, mixed, superactive, mesic Aridic Argixerolls) collected to a depth of 15 cm from the UNR Valley Road Field Lab field site, Reno NV. The soil type selected was used to simulate the growing conditions of these plants in their natural environment. The soil collected from the field was spread over a concrete surface to approximately 3 cm layer thick, air-dried for 10 days, and sifted to pass a 2-mm screen before each of the 54 tree pot (27.95 cm diameter × 60.96 cm height) containers (37.4 L volume) was filled with 20.4 kg of the air-dried soil. The soil was randomly sampled at the collection site to a depth of 15 cm and composited before soil test analysis was carried out at a commercial laboratory (A & L Western Agricultural Laboratories, Modesto, CA). The mean soil pH in water was 7.8, Olsen extractable P, K, Mg and Ca concentrations in the 0- to 15-cm depth soil sampled were 43, 376, 331, 1505 mg kg^−1^ soil and NO_3_-N was 3 mg kg^−1^ soil. One seedling was transplanted into each of the 54 pots representing each local populations and cultivars of the listed species in mid-November and remained in the pots for the duration of the study. Pots were spaced equally in an east to west direction on three greenhouse benches with two replications (rows) on each bench arranged in a randomized complete block design with a total of six replications of each sourced population used. Pots on each bench were rotated across benches monthly to minimize any variation in greenhouse temperature and humidity. No fertilizer or other soil amendments, and no supplemental light were provided to plants in this study to match closely their natural environment. These plants were watered twice weekly (Mondays and Fridays) using an automatic metered mist sprinkler system (Netafim USA, Fresno, CA) with a set run time of 15 minutes throughout the experimental period. The twice-weekly irrigation schedule was based on past observation in the greenhouse where plants wilt with a single watering schedule weekly for these native plants. Soil volume metric water content in each pot was measured using a Decagon ECH_2_O 5TM soil moisture and temperature sensor with a data logger (Decagon Devices, Inc., Pullman, WA) within five minutes after each irrigation run time was completed. The average tree pot soil volume metric water content was 0.274 m^3^/m^3^ after each irrigation interval throughout the study period. Greenhouse temperature was set to simulate the average monthly maximum (5:30 a.m. to 6:30 p.m.) and minimum temperature (6:30 p.m. to 5:29 a.m.) throughout the experimental period. Relative humidity in the greenhouse was maintained at 30% daily for the duration of the study.

### 4.3. Data Collected

Plant height (distance between soil surface of the pot to the collar of flag leaf) and leaf length (distance from ligule to the tip of the leaf blade) were measured twice (mid-season (15 March) and three days before harvest (20 June 2016)) during the experimental period. The duration from seed germination to floral initiation for each species was monitored and date recorded. Inflorescence length (distance from the bottom of the node to the tip of longest spike/panicle) was recorded twice (one month after floral initiation was observed in the greenhouse 19 May and the day before the seed and biomass harvest, 22 June 2016). In this greenhouse study, no flowering occurred for Sandberg bluegrass collections and this may have been the result of day length (affected by adjacent greenhouse bay with artificial lighting) or light intensity since no supplemental lighting was provided thus restricting floral induction of Sandberg bluegrass. Tiller counts (number of tillers per plant) were done three times, first at mid-April (14th), mid-May (19th), and two days before whole plant harvest (21 June 2016) to represent the average number of tillers per plant over the duration of the study. Shoot and root biomass were collected at the end of the experimental period (23 June 2016). Thereafter, shoot biomass was oven dried at 55 °C for 72 hours for dry matter (DM) determination in a forced-air oven.

### 4.4. Root Image Scanning and Analysis

The fresh roots from four plants for each treatment was removed carefully from pots, washed completely and placed in a cooler box prior to root scanning. Because of the size of these adult plants root system, each plant root system was segmented into smaller proportions (10-20 segments) before scanning. The segmented roots for each plant species were immersed in 4 mm of water in a 0.3- by 0.2-m Plexiglas tray and carefully separated using a toothpick to reduce overlapping. After which, a dual-scan optical scanner (Regent Instruments Inc. Ville de Québec, QC Canada) connected to a computer was then used to capture the root image of each plant species sample. The captured images were then analyzed using the WinRHIZO^TM^ software (Regent Instruments Inc. Ville de Québec, QC Canada) for root morphological traits namely, total root length (TRL), average root diameter (RD), and the root area volume (RAV). After root scanning, all roots were oven dried at 55 °C for 72 hours for dry matter (DM) determination in a forced-air oven. The root-to-shoot biomass ratio was computed by dividing the root biomass by shoot biomass of individual plant and reported as the proportion of root biomass in grams to every gram of shoot biomass. Specific root length (SRL) was calculated as the root length in meters divided by root biomass in grams (meters of root length per gram). Both the oven dried root and shoot biomass samples for each population were ground separately to pass a 1-mm screen using a UDY Cyclone Sample Mill (UDY Corporation, Fort Collins, CO). The ground shoot biomass of each sample was analyzed for the nutritive value parameters of crude protein (CP), acid detergent fiber (ADF) a measure of the plant component least digestible by livestock (includes; cellulose and lignin), neutral detergent fiber (NDF) the digestible and indigestible cell wall components remaining after the cell soluble were removed [92], acid detergent lignin (ADL), ash, and in vitro true digestibility (IVTD, 48 h). A micro-Kjeldahl technique was used to determine N concentration using a Kjeltech^TM^ 8200 Kjeldahl distillation unit (Foss North America, Inc. Eden Prairie, MN, USA). Crude protein concentration was calculated by multiplying N by 6.25. Goering and Van Soest [93] procedures (modified ANKOM system (ANKOM Technology, Macedon, NY)) were used to determine acid detergent fiber (ADF), neutral detergent fiber (NDF), and acid detergent lignin (ADL). The ash content was determined by placing samples in separate crucibles into a muffle furnace set at 500 °C for 3 h duration. A modified (ANKOM system) version of Tilley and Terry [94] was used to determine IVTD. The ground root samples were subsequently analyzed for carbon and nitrogen concentration using a LECO CN Analyzer (LECO Corporation, St Joseph, MI).

### 4.5. Data Analysis

Data collected were analyzed by fitting mixed models using PROC MIXED procedure in SAS [89]. Plant population source was treated as the fixed effect in the model and block (rep) was considered a random effect. Plant population traits means were different at *P* < 0.05 unless otherwise stated. The means separation for all traits of the two native bunchgrasses were determined using the Tukey’s test. Orthogonal contrasts were done for comparison between cultivar and the locally-sourced populations of Sandberg bluegrass and squirreltail. Pearson’s product–moment correlation coefficients among the measured parameters for shoot biomass, plant height, tiller number, and inflorescence length was conducted using the PROC CORR procedure of SAS [95] to help offer an explanation for the biomass trends of squirreltail populations observed in this study.

## 5. Conclusions

Approaches to ecosystem restoration in the Great Basin shrub steppe of the Western United States and globally, remains an enduring and widely debated topic. Overall, among the populations of the two native bunchgrasses (Sandberg bluegrass and Bottlebrush squirreltail), very few distinct differences in functional traits were evident in this study and for the few that occurred, they were all aboveground morphological traits. The functional traits that were obviously different among populations for either Sandberg bluegrass or Bottlebrush squirreltail were leaf length, plant height, and shoot biomass. In relation to forage nutritive value, ADF and IVTD were superior for Sandberg bluegrass cultivar relative to the local population, and NDF and ADL were lower for cultivar than local population of Squirreltail which are partial indicators of greater forage quality for the cultivated varieties of the two bunchgrasses relative to their wildland populations. However, for belowground functional traits, none of the morphological and chemical composition parameters appraised were distinctly different. Further, a key biomass production trait that is, the number of tillers per plant was similar among populations of both bunchgrasses. This indicates the closeness of these populations for both Sandberg bluegrass and Bottlebrush squirreltail at their adult stage and if the same results were to be obtained in common garden field experiments, it may reduce the fear of cultivar vigor, gene flow, and the notion of maladaptation of cultivars to these restoration ecozones. The results offered greater insights and emphasis for cultivar use when there is a scarcity in local seed source for restoration efforts. The traits expounded on in this study provide insights for building a unified framework approach among the different agencies and restoration practitioners to aid in plant assemblages for restoration success in the Great Basin and beyond.

## Figures and Tables

**Table 1:** Plant height, leaf length, tillers per plant, inflorescence length, root carbon, and nitrogen content of Sandberg bluegrass and Bottlebrush squirreltail populations grown under greenhouse conditions at the University of Nevada, Reno 2016.

Plant Species	Entry	Plant Height (cm)	Leaf Length (cm)	Tiller (plant^−1^)	Inflorescence Length (cm)	Root Carbon Content (g/plant)	Root Nitrogen Content (mg/plant)
Sandberg bluegrass	Button Point (LP)¶	20.5	13.2^c^	268	-	341.3	13.4
Sandberg bluegrass	Panther Valley (LP)	23.4	16.3^ab^	252	-	328.5	14.2
Sandberg bluegrass	Winnemucca Mountain (LP)	23.1	15.5^abc^	224	-	287.5	12.8
Sandberg bluegrass	Hanford (CV)	23.6	18.4^a^	312	-	383.3	14.6
Sandberg bluegrass	Mountain Home (CV)	22.9	15.0^bc¶^	258	-	385.7	17.7
SEM†		1.7	1.1	33	-	97.4	4.5
*P* value‡		0.714	0.032	0.451	-	0.899	0.920
Squirreltail	George St. Sonoma (LP)	71.0^a¶^	17.3	150	8.9^c^	89.3b	3.7b
Squirreltail	Grass Valley (LP)	71.6^a^	15.9	135	11.4^bc^	120.7ab	5.2ab
Squirreltail	Toe Jam Creek (CV)	43.0^b^	20.4	135	13.2^ab^	237.9a	9.4a
Squirreltail	Vale (CV)	37.4^b^	17.1	152	16.3^a^	160.5ab	6.4ab
SEM†		3.9	1.1	15	1.2	48.8	1.9
*P* Value‡		<0.001	0.064	0.482	0.005	0.076	0.094

†SEM = standard error of the mean, ‡ *P* value = to compare treatment means within column, and ¶ within columns, means followed by same lowercase superscripts letter are not different (*P* > 0.05). ¶; in parentheses, LP indicates local population, and CV indicates cultivar.

**Table 2:** Plant species and their classifications used in a greenhouse pot experiment to characterized adult functional traits of native bunchgrasses commonly used in the restoration of the Great Basin shrub steppe ecosystem in the Western United States.

Plant Species	Classification	Seed Source	Site Elevation (m)	Average Annual Precipitation (mm)	Seed Collection Reference Number	Latitude	Longitude	Number of Plants Sampled and Area in Parentheses	Soil Type
*Poa secunda*	Wildland	Button Point	1340	203.2	NV020-02	41° 1′ 22.92” N	117° 35′ 40.07′ W	10,000 (20 acres)	Silt
*Poa secunda*	Wildland	Panther Valley	1495	188.0	NV020-03	40° 32′ 51.15” N	117° 35′ 48.16” W	Not available	Droughty loam
*Poa secunda*	Wildland	Winnemucca Mountain	1372	208.3	NV020-04	41° 02′ 26.10” N	117° 43′ 28.57” W	10,000 (50 acres)	Sandy loam
*Poa secunda*	Cultivar	Hanford†							
*Poa secunda*	Cultivar	Mountain Home‡							
*Elymus elymoides*	Wildland	George St. Sonoma	1368	208.3	NV020-01	40° 44′ 53.61” N	117° 43′ 19.33” W	100 (20 acres)	Droughty loam
*Elymus elymoides*	Wildland	Grass Valley	1485	208.3	NV020-07	40° 29′ 29.04” N	117° 36′ 12.83”	1000 (3 acres)	Droughty loam
*Elymus elymoides*	Cultivar	Toe Jam Creek							
*Elymus elymoides*	Cultivar	Vale§							

† Hanford source Sandberg bluegrass is a source identified release from L&H Seeds in Connell, Washington. The original seed source was collected from Hanford, Washington from an area receiving approximately 152 mm precipitation per annum [87].

‡Mountain Home Germplasm; accession numbers RMRS B53, W6 39684, PI 660255. First collected in 1997 in Owyhee County, Idaho at an elevation of 900 km [30].

¶ Toe Jam Creek; *Elymus elymoides* subsp. *californicus* germplasm (Reg.no. GP-89, PI 531604) was released on September 4, 2003. Toe Jam Creek was collected in Northwestern Elko, county, Nevada, USA. The elevation at the site is 1829 m with average annual precipitation of 312 mm [90].

§ Vale; The origin of the cultivar is Malheur, Oregon, USA and it is a blend of *E. elymoides* ssp. *californicus* and “ssp. C.” [91].
